# Small-Pox

**Published:** 1848-01

**Authors:** 


SMALL-POX.

It is reported that several members of the Indiana Legislature having contracted the small-pox at the seat of government of that State, the Legislature has adjourned to meet in February.

A very curious account of the occurrence of the disease, or something simulating it, in the sheep, is contained in our *Record* department of the present number.

